# Somatotopic Representation of Second Pain in the Primary Somatosensory Cortex of Humans and Rodents

**DOI:** 10.1523/JNEUROSCI.3654-17.2018

**Published:** 2018-06-13

**Authors:** Q.Q. Jin, G.Q. Wu, W.W. Peng, X.L. Xia, L. Hu, G.D. Iannetti

**Affiliations:** ^1^CAS Key Laboratory of Mental Health, Institute of Psychology, Beijing, 100101, China,; ^2^Department of Psychology, University of Chinese Academy of Sciences, Beijing 100049, China,; ^3^Brain Function and Psychological Science Research Center, Shenzhen University, Shenzhen 518060, China,; ^4^Department of Neuroscience, Physiology and Pharmacology, University College London, London WC1E 6BT, United Kingdom, and; ^5^Istituto Italiano di Tecnologia, 16163 Genova, Italy

**Keywords:** C fibers, electrocorticography (ECoG), electroencephalography (EEG), multispecies investigations, pain, primary somatosensory cortex (S1)

## Abstract

There is now compelling evidence that selective stimulation of Aδ nociceptors eliciting first pain evokes robust responses in the primary somatosensory cortex (S1). In contrast, whether the C-fiber nociceptive input eliciting second pain has an organized projection to S1 remains an open question. Here, we recorded the electrocortical responses elicited by nociceptive-specific laser stimulation of the four limbs in 202 humans (both males and females, using EEG) and 12 freely moving rats (all males, using ECoG). Topographical analysis and source modeling revealed in both species, a clear gross somatotopy of the unmyelinated C-fiber input within the S1 contralateral to the stimulated side. In the human EEG, S1 activity could be isolated as an early-latency negative deflection (C-N1 wave peaking at 710–730 ms) after hand stimulation, but not after foot stimulation because of the spatiotemporal overlap with the subsequent large-amplitude supramodal vertex waves (C-N2/P2). In contrast, because of the across-species difference in the representation of the body surface within S1, S1 activity could be isolated in rat ECoG as a C-N1 after both forepaw and hindpaw stimulation. Finally, we observed a functional dissociation between the generators of the somatosensory-specific lateralized waves (C-N1) and those of the supramodal vertex waves (C-N2/P2), indicating that C-fiber unmyelinated input is processed in functionally distinct somatosensory and multimodal cortical areas. These findings demonstrated that C-fiber input conveys information about the spatial location of noxious stimulation across the body surface, a prerequisite for deploying an appropriate defensive motor repertoire.

**SIGNIFICANCE STATEMENT** Unmyelinated C-fibers are the evolutionarily oldest peripheral afferents responding to noxious environmental stimuli. Whether C-fiber input conveys information about the spatial location of the noxious stimulation to the primary somatosensory cortex (S1) remains an open issue. In this study, C-fibers were activated by radiant heat stimuli delivered to different parts of the body in both humans and rodents while electrical brain activity was recorded. In both species, the C-fiber peripheral input projects to different parts of the contralateral S1, coherently with the representation of the body surface within this brain region. These findings demonstrate that C-fiber input conveys information about the spatial location of noxious stimulation across the body surface, a prerequisite for deploying an appropriate defensive motor repertoire.

## Introduction

Because of the different conduction velocity of Aδ (∼15 m/s) and C (∼1 m/s) afferents, a single and intense cutaneous nociceptive stimulus elicits a typical double sensation: an initial Aδ-fiber-related pricking “first” pain is followed by a C-fiber-related burning “second” pain (Lewis and Pochin, 1937; Basbaum and Bushnell, 2009).

In humans, the brain electrical responses elicited by laser heat pulses that concomitantly activate Aδ- and C-fiber endings in the superficial skin layers (laser-evoked brain potentials, LEPs) (Bromm and Treede, 1984) show clear deflections at latencies compatible with the conduction velocity of Aδ-fibers (Aδ-LEPs or late LEPs; Treede et al., 1988). The largest deflection is a negative–positive complex (N2-P2) maximal at the scalp vertex, peaking at ∼200–350 ms when stimulating the hand dorsum (Bromm and Treede, 1984). This N2-P2 complex is functionally similar to the vertex potentials elicited by sudden stimuli belonging to non-nociceptive sensory modalities (Mouraux and Iannetti, 2009) and largely reflects supramodal saliency-related neural processes, possibly consequent to the detection of behaviorally relevant changes in the sensory environment (Downar et al., 2000). The N2-P2 vertex complex is preceded by a smaller negative deflection (N1), which, when stimulating the hand dorsum, peaks at ∼160 ms and is maximal over the central temporal region contralateral to the stimulated side (Tarkka and Treede, 1993). The early contralateral N1 wave of the Aδ-LEPs reflects somatosensory-specific activity more related to the magnitude of the incoming nociceptive input (Lee et al., 2009). This somatosensory-specific activity is likely to subserve the high spatial acuity for first pain (Mancini et al., 2013), as indicated by the existence of fine-grained maps of Aδ nociceptive input to individual digits in the human primary somatosensory cortex (S1) (Mancini et al., 2012).

In contrast, the EEG deflections related to the activation of C-fibers (C-LEPs or ultralate LEPs; Iannetti et al., 2003) are more difficult to detect. C-LEPs have initially been suggested to appear only when the concomitant activation of Aδ-fibers is avoided or reduced (Bromm and Treede, 1983; Bragard et al., 1996; Mouraux et al., 2003) because the low intensity and high temporal predictability of the C-fiber input make it less salient than the preceding Aδ input (Iannetti et al., 2008; Mouraux and Iannetti, 2009; Ronga et al., 2013). However, we recently showed that by applying advanced signal processing techniques (e.g., peak alignment and time-frequency decomposition) and using optimal stimulus parameters (e.g., delivering a high number of stimuli of multiple energies within a small skin territory), the N2-P2 vertex complex can be reliably detected in single-subject C-LEPs, even when preceded by Aδ-LEPs (Hu et al., 2014b).

Different from humans, Aδ afferents are virtually insensitive to heat in rodents (Lynn and Shakhanbeh, 1988; Shim et al., 2005). Therefore, the same laser heat pulses that coactivate Aδ and C-fiber endings in primates (Treede et al., 1998; Magerl et al., 1999) almost uniquely activate C-fiber afferents when delivered to the rat skin (Sikandar et al., 2013; Hess, 2015; Hu et al., 2015). Therefore, both pain-related behavior and central responses elicited by laser pulses in rats occur at latencies only compatible with the activation of C-fiber primary afferents (Sikandar et al., 2013; Xia et al., 2016).

Here, we tested whether the C-fiber input has a somatotopically organized projection to the S1 in humans and rodents. We recorded 64-channel EEG responses elicited by laser stimulation of the four limbs in 202 healthy human participants. We also recorded 14-channel ECoG responses elicited by the same laser stimulation of the four paws in 12 awake and freely moving rats. We used topographical analysis and source modeling both at group and single-subject level to test the working hypothesis. Furthermore, the concomitant presence of both Aδ-LEPs and C-LEPs in human recordings allowed us to perform a comprehensive correlation analysis of the different deflections. With this analysis, we explored the functional relationship between somatosensory-specific and supramodal generators of the brain responses evoked by C-fiber input.

## Materials and Methods

### 

#### Experiment 1: Human EEG during hand stimulation

##### Subjects.

EEG data were collected from 107 healthy subjects (67 females) aged 21.6 ± 1.8 years (mean ± SD, range = 18–26 years). All subjects gave their written informed consent and were paid for their participation. The local ethics committee approved the experiment procedures.

##### Nociceptive stimulation.

Nociceptive-specific radiant heat stimuli were generated by an infrared neodymium yttrium aluminum perovskite (Nd:YAP) laser with a wavelength of 1.34 μm (Electronical Engineering). At this wavelength, laser pulses activate epidermal nociceptive terminals belonging to Aδ and C primary nociceptors directly (Iannetti et al., 2003). Laser pulses were delivered at the dorsum of the left hand (LH) and right hand (RH) on a squared area (5 × 5 cm^2^) defined before the beginning of the experimental session. An He–Ne laser pointed to the area to be stimulated. The laser pulse was transmitted via an optic fiber and its diameter was set at ∼7 mm (∼38 mm^2^) by focusing lenses. The duration of the laser pulse was 4 ms. To allow for passive skin cooling and avoid nociceptor fatigue or sensitization, the laser beam target was shifted by ∼1 cm in a random direction after each stimulus. After each stimulus, subjects were instructed to rate the intensity of perceived pain on a 0–10 numerical rating scale (NRS), with 0 standing for “no sensation” and 10 standing from “pain as bad as it could possibly be.”

##### Experimental design.

Before the EEG data collection, for each subject, we identified the 4 laser energies that evoked subjective pain ratings of ∼2, ∼4, ∼6, and ∼8 on the 0–10 NRS by increasing the stimulus energy in steps of 0.25 J until the target rating was reached. Across the group, these energies were as follows: E1, 2.0 ± 0.2 J; E2, 2.7 ± 0.3 J; E3, 3.4 ± 0.3 J; and E4, 4.1 ± 0.4 J. The experiment consisted of two blocks. In each block, laser stimuli were delivered to the dorsum of either the left hand (LH) or the right hand (RH). In each block, 10 laser pulses of each stimulus energy were delivered, for a total of 40 pulses per stimulation side (80 pulses in total). The order of blocks was counterbalanced across subjects and the order of stimulus energies was pseudorandomized. The surface temperature of the stimulated hand dorsum was measured before each block using an infrared thermometer, to ensure that the difference between the two blocks was never >1°C (Iannetti et al., 2004; Baumgärtner et al., 2005). This temperature was 33.3 ± 1.1°C and 33.4 ± 1.1°C before each block, respectively (*t*_(106)_ = −0.87, *p* = 0.39, paired-samples *t* test). An auditory tone was delivered randomly between 3 and 6 s after the laser stimulation to prompt subjects to rate the intensity of pain perception elicited by the laser stimulus on the same 0–10 NRS. The interstimulus interval varied randomly between 11 and 15 s using a rectangular distribution.

##### EEG recording.

Subjects were seated in a comfortable chair in a silent, temperature-controlled room and wore protective goggles. They were asked to focus their attention on the stimuli, relax their muscles, and keep their eyes open and gaze slightly downward. EEG data were recorded using 64 Ag-AgCl electrodes placed on the scalp according to the International 10–20 system, using the nose as reference (Brain Products; pass band: 0.01–100 Hz; sampling rate: 1000 Hz). Electrode impedances were kept <10 kΩ. To monitor ocular movements and eye blinks, EOG signals were simultaneously recorded from two surface electrodes, one placed over the lower eyelid and the other placed 1 cm lateral to the outer corner of the orbit.

##### EEG data analysis.

EEG data were processed using EEGLAB (Delorme and Makeig, 2004), an open source toolbox running in the MATLAB environment (The MathWorks). Continuous EEG data were band-pass filtered between 1 and 30 Hz. EEG epochs were extracted using a window analysis time of 3000 ms (1000 ms prestimulus and 2000 ms poststimulus) and baseline corrected using the prestimulus interval. Based on visual inspection, trials contaminated by artifacts due to gross movements were removed. In addition, trials contaminated by eye blinks and movements were corrected using an independent component analysis (ICA) algorithm (Delorme and Makeig, 2004). In all datasets, these independent components (ICs) had a large EOG channel contribution and a frontal scalp distribution. After preprocessing, EEG data were rereferenced to the common average.

Across four stimulus energies (E1–E4), epochs belonging to the same stimulation side (LH or RH) were averaged time-locked to stimulus onset, thus yielding two average waveforms for each subject. Peak latencies and amplitudes of Aδ-N2, Aδ-P2, C-N2, and C-P2 waves were measured from single-subject average waveforms (Cz-average). Aδ-N2 and Aδ-P2 waves were defined as the most negative and positive deflections between 150 and 500 ms after stimulus onset (Kunde and Treede, 1993; Tu et al., 2016). C-N2 and C-P2 waves were defined as the most negative and positive deflections between 700 and 1300 ms after stimulus onset (Hu et al., 2014b). Single-subject average waveforms were subsequently averaged to obtain group-level waveforms. Group-level scalp topographies at the peak latency of the Aδ-N2, Aδ-P2, C-N2, and C-P2 waves were computed by spline interpolation.

##### Statistical analysis.

To explore the spatial differences between the response elicited by the stimulation of the LH and RH, we plotted the scalp topographies of the earliest part of Aδ-LEPs (i.e., from 150–170 ms, corresponding to the Aδ-N1) and of C-LEPs (i.e., from 710–730 ms, corresponding to the C-N1), in steps of 10 ms. To test whether these earliest responses were lateralized with respect to the stimulated hand, we compared single-subject LEP waveforms elicited by the stimulation of the LH and RH using the following procedure (Hu et al., 2014a). First, for each subject and each stimulation side, LEP waveforms were normalized and expressed as *Z*-values by subtracting from each time point the mean amplitude of the waveform and then dividing the resulting value by the SD of the waveform amplitude. Second, a point-by-point paired-samples *t* test was used to compare LEP responses elicited by stimulation of the LH and RH dorsum, which yielded a time course of *p*-values representing the significance of the difference between LEPs elicited by LH and RH stimulation for each electrode. Third, to account for multiple comparisons of different time points and electrodes, a false discovery rate (FDR) (Benjamini and Hochberg, 1995) procedure was used to correct the significance level (*p*-value). Fourth, single-subject difference LEP waveforms (LH − RH) were calculated and averaged across subjects, to display the group-level difference of LEPs elicited by stimulation of the LH and RH dorsum.

To estimate the locations of Aδ-N1 and C-N1 sources, group-level LEP waveforms were imported in Brain Electrical Source Analysis Software (BESA 5.3; MEGIS Software). Sources of LEP waveforms were modeled using equivalent current dipoles from a spatiotemporal source model (Scherg and Ebersole, 1993; Lopes da Silva, 2004). Based on findings of several previous studies (e.g., Garcia-Larrea et al., 2003; Valentini et al., 2012), a four-dipole model was used. Dipole configurations were calculated within a realistic head model and estimated according to the best correspondence between the recorded and estimated scalp distribution. The ability of the estimated dipoles to explain satisfactorily the recorded scalp topography was expressed as the signal residual variance; that is, the percentage of the scalp data that cannot be explained by the fitted dipoles. For each dipole, the location, orientation, and time course were extracted and source locations were transformed to normalized Talairach space.

Because of the contralateral scalp distribution of the early LEP activity (i.e., Aδ-N1 and C-N1), to measure the amplitudes of Aδ-N1 and C-N1 waves, we used a bipolar montage consisting of the contralateral central electrode referenced to Fz (Cc-Fz) (Hu et al., 2010; Valentini et al., 2012). From the single-subject Cc-Fz rereferenced LEP waveform averaged across trials and stimulus energies, peak latencies and amplitudes of Aδ-N1 and C-N1 waves were measured manually for each subject (if a clear response could not be detected, then default values, “NaN,” were assigned). Aδ-N1 and C-N1 were defined as the negative deflections preceding the Aδ-N2 and C-N2 waves, respectively (Tarkka and Treede, 1993; Valentini et al., 2012). Only unique and clear negative deflections preceding the Aδ-N2 (C-N2) waves were classified as Aδ-N1 (C-N1) responses [Aδ-N1: 81.3% of subjects (both LH and RH); C-N1: 72.9% (LH) and 75.7% (RH)]. Aδ-N1 (C-N1) responses were defined as absent if the Aδ-N1 (C-N1) amplitude was positive or if a clear negative deflection preceding the Aδ-N2 (C-N2) wave could not be identified (Hu et al., 2010). Single-subject LEP waveforms (Cc-Fz) were subsequently averaged to obtain group-level rereferenced waveforms. Group-level scalp topographies at the peak latencies of Aδ-N1 and C-N1 waves were computed by spline interpolation.

To explore the functional relationship between the C-N1 and the other LEP waves, latencies and amplitudes of C-N1 wave were correlated with those of other LEP waves (i.e., Aδ-N1, Aδ-N2, Aδ-P2, C-N2, and C-P2 waves) across subjects using Pearson's correlation analysis. To account for multiple comparisons, *p*-values were adjusted using FDR correction.

Furthermore, to assess the dependence of Aδ-N1 and C-N1 amplitudes on stimulus energy, we calculated the averaged LEP waveform for each level of stimulus energy, thus obtaining four average waveforms (E1–E4) for each stimulated hand (LH and RH). Peak amplitudes of Aδ-N1 and C-N1 waves at each stimulus energy were measured from the waveforms (Cc-Fz) of each subject. Aδ-N1 and C-N1 amplitudes were compared statistically using a two-way repeated-measures ANOVA, with stimulation site (two levels: LH and RH) and stimulus energy (four levels: E1–E4) as within-subject factors. When significant, *post hoc* comparisons were performed using paired-samples *t* tests.

To assess the dependence of Aδ-N1 and C-N1 amplitudes on perceived pain intensity, we classified single trials in four categories (I1–I4) according to the subjective pain ratings. This was achieved by first rescaling the ratings of each subject between 0 and 100, defining the smallest pain rating as 0 and the largest pain rating as 100 (Zhang et al., 2012). For each subject and each stimulated hand (LH and RH), trials were then classified in four categories (I1 = ≤25, I2 = >25 and ≤50, I3 = >50 and ≤75, and I4 = >75). EEG trials belonging to each category were averaged together, thus yielding four average waveforms for each subject and stimulated hand. Finally, peak amplitudes of Aδ-N1 and C-N1 waves of each of the four categories were measured from the average waveforms (Cc-Fz) of each subject. Aδ-N1 and C-N1 amplitudes were compared statistically using a two-way repeated-measures ANOVA, with stimulation site (two levels: LH and RH) and pain intensity (four levels: I1-I4) as within-subject factors. When significant, *post hoc* comparisons were performed using paired-samples *t* tests.

Because Aδ-N1 and C-N1 waves overlap spatially and temporally with the subsequent N2-P2 complex, the signal-to-noise ratio of Aδ-N1 and C-N1 activities can be largely enhanced if the contamination of the N2-P2 complex is removed from the LEP responses (Hu et al., 2010). The removal of N2-P2 related activities can be achieved by performing an additional ICA and removing N2 and P2-related ICs, which are easily identified because of the high signal-to-noise ratio of the N2-P2 complex (Mouraux and Iannetti, 2008). Therefore, to isolate Aδ-N1 and C-N1 activities, LEP responses were decomposed into a series of ICs, each having a maximally independent time course and a fixed scalp topography. N2- and P2-related ICs were identified and removed, thus generating a new set of EEG data devoid of ICs representing N2-P2 related activities. As described in Hu et al. (2010), ICs were classified as “N2-P2 related” only if they satisfied the following three criteria: (1) reflecting neural activity elicited by the laser stimulus (this was tested by normalizing the time course of the power of each IC as the SD from the mean of the prestimulus interval, −1000–0 ms, expressed as *Z* score, and checking if the average *Z* score within 0 to +1500 ms poststimulus interval was larger than 5); (2) having peaks with a latency between 150 and 500 ms (Aδ-N2/P2) and/or between 700 and 1300 ms (C-N2/P2); and (3) having a scalp topography centrally distributed and maximal at the vertex. After Aδ-N2/P2- and C-N2/P2-related ICs were removed using this approach, Aδ-N1 and C-N1 peaks were detected using the bipolar montage Cc-Fz, and their scalp topographies were plotted at the respective peak latencies.

#### Experiment 2: Human EEG during foot stimulation

##### Subjects.

EEG data were collected from a different group of 95 healthy new subjects (50 females) aged 21.6 ± 1.7 years (mean ± SD, range = 18–25 years). All subjects gave their written informed consent and were paid for their participation. The local ethics committee approved the experimental procedures.

##### Nociceptive stimulation and experimental design.

Nociceptive stimulation and experimental design were identical to Experiment 1, with the exception that laser stimuli were delivered on the dorsum of the left and right foot, and that stimulation energies were as follows: E1: 2.5 J; E2: 3 J; E3: 3.5 J; and E4: 4 J.

##### EEG recording *and* data analysis.

EEG recording and data analysis were identical to Experiment 1 with two exceptions due to the fact that nociceptive stimuli were delivered to the foot. First, the C-N2 and C-P2 were defined as the most negative and positive deflections between 1000 and 1700 ms after stimulus onset. Second, it was not possible to measure N1 amplitude, which is notoriously difficult to isolate after foot stimulation because of the spatial and temporal overlap with the N2 wave.

#### Experiment 3: Rat ECoG during stimulation of the four paws

##### Subjects.

The experiment was conducted on 12 adult male Sprague Dawley rats weighing 300–400 g (Xia et al., 2016). Rats were fed *ad libitum* with water and food and were housed in separate cages under temperature- and humidity-controlled conditions. They were kept in a 12 h day/night cycle (lights on from 08:00–20:00). All experimental procedures adhered to the guidelines for animal experimentation.

##### Nociceptive stimulation and experimental design.

Surgical procedures and electrode coordinates have been described previously (Hu et al., 2015). After surgery, rats were kept in their cages for at least 7 d before the collection of ECoG data. During the ECoG data collection, rats were placed into a plastic chamber (length × width × height: 30 × 30 × 40 cm^3^), within which they could move freely. The same Nd:YAP laser stimuli used in human experiments were delivered on the rats' paws through holes (5 mm diameter) on the floor of the chamber when the animal was spontaneously still. Ten laser pulses were delivered to each of the four stimulation sites (left forepaw, right forepaw, left hindpaw, and right hindpaw) using five stimulus energies (E1′–E5′ from 1–4 J in steps of 0.75 J) for a total of 200 pulses. The order of stimulation sites and stimulus energies was pseudorandomized, the interstimulus interval was never <30 s, and the laser target was changed after each stimulus to avoid nociceptor fatigue or sensitization. Animals were video-recorded throughout the experiment and pain-related behaviors elicited by the laser stimuli were quantified using a validated 0–4 scale where 0 is immobility; 1 is head turning, including shaking or elevating the head; 2 is flinching; 3 is withdrawal involving paw retraction from the nociceptive stimulus; and 4 is licking the stimulated body territory and whole-body movement (Fan et al., 1995, 2009). White noise was played throughout the experiment to avoid the activation of the auditory system by laser-generated ultrasounds and thereby uniquely record the cortical responses related to the activation of the nociceptive system (Xia et al., 2016).

##### ECoG recording and data analysis.

ECoG recording and analysis were virtually identical to Experiment 1. To explore the spatial differences between the response elicited by the stimulation of the left and right paws, we plotted the scalp topographies of the earliest part of C-LEPs (i.e., from 110–130 ms for forepaw stimulation and from 210–230 ms for hindpaw stimulation, corresponding to the C-N1) in steps of 10 ms. To test whether these earliest responses were lateralized with respect to the stimulated paw, we compared single-subject LEP waveforms elicited by the stimulation of the left and right paws using the same procedure of Experiments 1 and 2.

To minimize the influence of widespread activities and enhance spatially discrete activities like the C-N1 wave, we average referenced the data (Xia et al., 2016). We measured the peak latency and amplitude of the C-N1 waves in single-subject LEP waveforms averaged across trials and stimulus energies. Importantly, there was a clearly detectable C-N1 response in all animals. The C-N1 wave was optimally detected from electrode RFR (after left forepaw stimulation), LFL (after right forepaw stimulation), PR1 (after left hindpaw stimulation), and PL1 (after right hindpaw stimulation). Single-subject rereferenced waveforms were subsequently averaged to obtain group-level waveforms. Group-level scalp topographies at the peak latency of the C-N1 wave were computed by spline interpolation.

##### Statistical analysis.

To assess the dependence of C-N1 amplitudes on stimulus energy, we first calculated the average LEP waveform for each level of stimulus energy, thus yielding five average waveforms (E1′–E5′) for each of the four stimulation sites. Peak amplitude of the C-N1 wave at each stimulus energy was measured in each subject. C-N1 amplitudes were compared using a two-way repeated-measures ANOVA, with stimulation site (four levels: left and right forepaw and hindpaw) and stimulus energy (five levels: E1′–E5′) as within-subject factors. When significant, *post hoc* pairwise comparisons were performed using paired-samples *t* tests.

## Results

### Experiments 1 and 2: Human EEG

#### Waveforms and topographies of the early part of human C-LEPs

**Figure 1.**
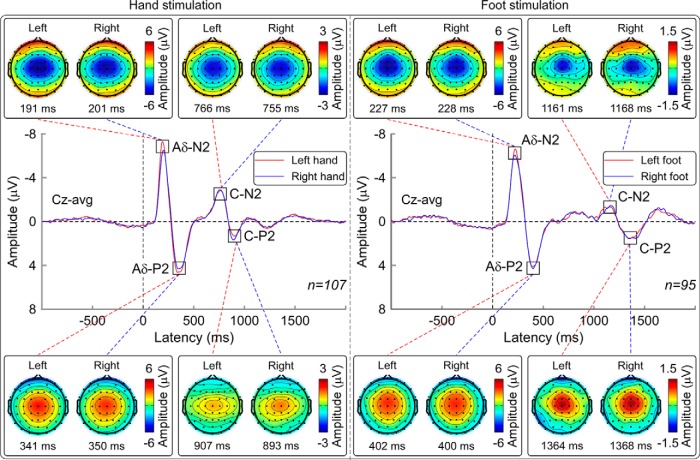
Grand average LEP waveforms and scalp topographies of Aδ-N2, Aδ-P2, C-N2, and C-P2 waves. LEP responses were elicited by hand stimulation (left) and foot stimulation (right), on the left side (red waveforms) and right side (blue waveforms). Data were collected from 64 electrodes in 107 subjects (hand stimulation) and 95 subjects (foot stimulation). Displayed signals were recorded from the vertex (Cz vs average reference). Scalp topographies are displayed at the peak latency of Aδ-N2, Aδ-P2, C-N2, and C-P2 waves.

Figure 1 shows the grand average human LEP waveforms at Cz and the scalp topographies at the peak latencies of Aδ-N2, Aδ-P2, C-N2, and C-P2 waves for both hand and foot stimulation. Regardless of where the stimulus was delivered, Aδ-N2 and C-N2 waves were always maximal at the vertex and extended bilaterally toward the temporal regions, whereas Aδ-P2 and C-P2 waves were more centrally distributed (Fig. 1), as also described in previous studies (Kunde and Treede, 1993; Mouraux and Iannetti, 2009; Hu et al., 2014b). Group-level latencies and amplitudes of the main peaks of both the Aδ-LEPs and C-LEPs are reported in Table 1.

**Table 1.** Latency and amplitude of LEP waves elicited by left and RH stimulation

	LH stimulation (mean ± SEM)		RH stimulation (mean ± SEM)	
	Latency (ms)	Amplitude (μV)	Latency (ms)	Amplitude (μV)
Aδ-N1	167 ± 3	−5.2 ± 0.3	173 ± 2	−4.3 ± 0.3
Aδ-N2	202 ± 2	−8.6 ± 0.4	206 ± 3	−7.8 ± 0.3
Aδ-P2	355 ± 4	6.1 ± 0.3	364 ± 4	5.7 ± 0.3
C-N1	736 ± 6	−3 ± 0.2	737 ± 6	−2.8 ± 0.2
C-N2	762 ± 5	−4.8 ± 0.2	765 ± 5	−4.9 ± 0.2
C-P2	886 ± 5	2.7 ± 0.2	890 ± 5	2.3 ± 0.2

Aδ-N2, Aδ-P2, C-N2, and C-P2 waves were measured at Cz-average.

Aδ-N1 and C-N1 waves were measured at Cc-Fz.

**Figure 2.**
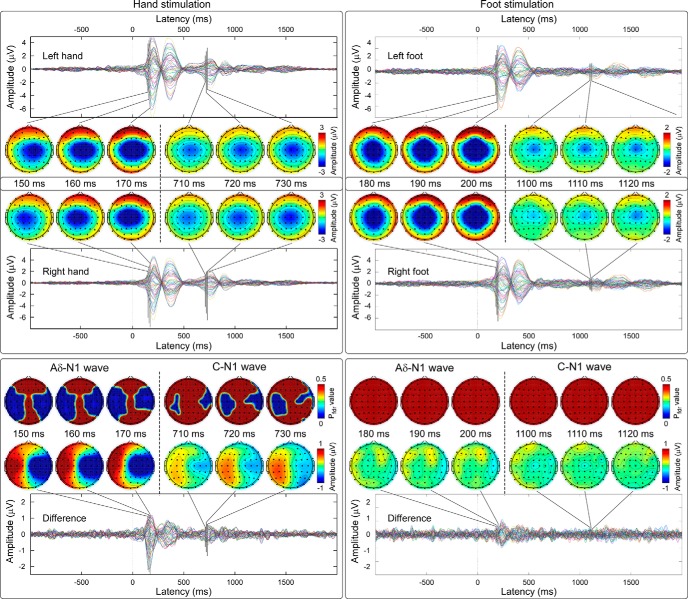
Topographies and statistical comparisons of the earliest part of Aδ- and C-LEPs elicited by left and right stimulation of hand and foot. Top, Grand averages from all electrodes are plotted in different colors and superimposed. Series of scalp topographies of the earliest part of Aδ-LEPs and C-LEPs (Aδ-LEP: 150–170 ms, hand; 180–200 ms, foot; C-LEP: 710–730 ms, hand; 1100–1120 ms, foot) are displayed at 10-ms intervals. Note that the scalp topographies of the earliest part of the Aδ-LEPs and C-LEPs elicited by hand stimulation show a negativity contralateral to the stimulated side, whereas the scalp topographies of the earliest part of the Aδ-LEPs and C-LEPs elicited by foot stimulation are centrally distributed. This observation is compatible with the somatotopical organization of the primary somatosensory cortex. Bottom, Grand averages and scalp topographies of the difference waveforms obtained by subtracting the LEPs elicited by right and left stimulation for hand (left) and foot (right). The top row shows the scalp topography of the statistical comparisons between left and right LEPs at the Aδ-N1 and C-N1 latencies. The earliest part of both Aδ-LEPs and C-LEPs elicited by hand stimulation shows significant differences (*p*_FDR_ < 0.05) localized bilaterally on the electrodes overlying the hand area in the primary somatosensory cortices. In contrast, the earliest part of both Aδ-LEPs and C-LEPs elicited by the stimulation of the left and right foot was not significantly different.

The top panels of Figure 2 shows the same grand average LEP waveforms, but from all recorded electrodes, in response to hand (left) and foot (right) stimulation. After hand stimulation, the scalp topographies of the earliest activity of both the Aδ-LEPs (150–170 ms) and the C-LEPs (710–730 ms) displayed a clear negative maximum on the central electrodes overlying the hemisphere contralateral to the stimulation side. In contrast, after foot stimulation, the scalp topographies of the earliest activity in both Aδ-LEPs (180–200 ms) and C-LEPs (1100–1120 ms) were centrally distributed. The bottom panels of Figure 2 show the grand average of the difference between LEP waveforms elicited by the stimulation of the left and right side, as well as series of scalp topographies in the earliest part of both Aδ-LEPs and C-LEPs (Aδ-LEP: 150–170 ms, hand; 180–200 ms, foot; C-LEP: 710–730 ms, hand; 1100–1120 ms, foot). In these time intervals, Aδ-LEPs and C-LEPs were significantly different when elicited by left and right hand stimulation (*p*_FDR_ < 0.05), but not different when elicited by left and right foot stimulation. The differences observed after hand stimulation occurred at the latencies of the Aδ-N1 and of the C-N1 waves, and were localized bilaterally on the electrodes overlying the hand area in the S1 (Fig. 2).

#### Source analysis of the C-N1 waves elicited by hand and foot stimulation

**Figure 3.**
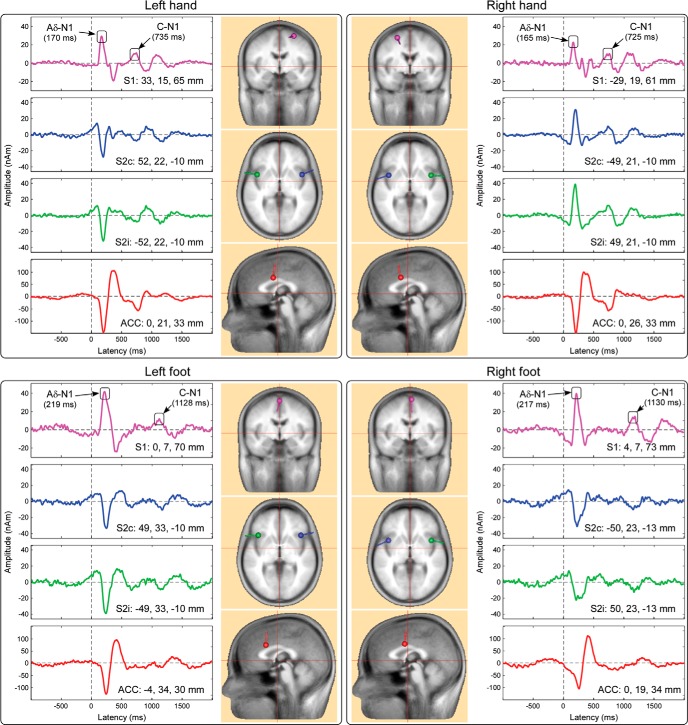
Dipolar sources of group average scalp LEP waveforms elicited by hand (top) and foot (bottom) stimulation. LEP responses were explained by four equivalent dipoles, located in the S1 (pink), the bilateral S2 (green and blue), and the ACC (red). Source time courses are displayed in the lateral part of each panel. Note that the spatial locations of S2 and ACC dipoles are similar regardless of stimulated side. In contrast, the spatial location of the S1 dipole varies according to the stimulated side, but only when stimuli are delivered to the hand (top): it was located in the right S1 when the LH was stimulated and vice versa. Note also that the peaks of the S1 source time course occur at the latency of the Aδ-N1 and C-N1 waves.

Time courses and locations of the dipolar sources of LEPs are shown in Figure 3. The source explaining the time window of the Aδ-N1 and C-N1 waves was located in the contralateral S1, whereas the sources explaining the time window of the Aδ-N2/P2 and C-N2/P2 complex were located in the bilateral operculo-insular cortices (S2 and/or insula) and in the anterior cingulate cortex (ACC). Although the location of bilateral S2/insula and ACC were almost identical regardless of stimulated site (hand or foot) or stimulated side (right or left), the generator of the N1 elicited by hand stimulation was always *contralateral* to the stimulated side (Fig. 3, top): in the right S1 when the LH was stimulated, and in the left S1 when the RH was stimulated. Importantly, this was not the case when considering the earliest part of the Aδ- and C-LEPs elicited by foot stimulation (Fig. 3, bottom): regardless of whether the right or the left foot was stimulated, the S1 generator was always located on the midline in the foot area of the S1. At all stimulated sites, the time course of the S1 source activity clearly showed both Aδ-N1 and C-N1 waves. The residual variance within the Aδ-N1 time window (150–170 ms, hand; 180–200 ms, foot) was 2.8% and 1.4% for LH and RH stimulation, and 3.1% and 6.1% for left and right foot stimulation. The residual variance within the C-N1 time window (710–730 ms, hand; 1110–1130 ms, foot) was 3.9% and 2.3% for LH and RH stimulation, and 9.3% and 5.1% for left and right foot stimulation. These results indicate that dipoles in S1 explained satisfactorily the scalp topography of both the Aδ-N1 and the C-N1 peaks.

#### Isolation of the C-N1 wave elicited by hand stimulation

**Figure 4.**
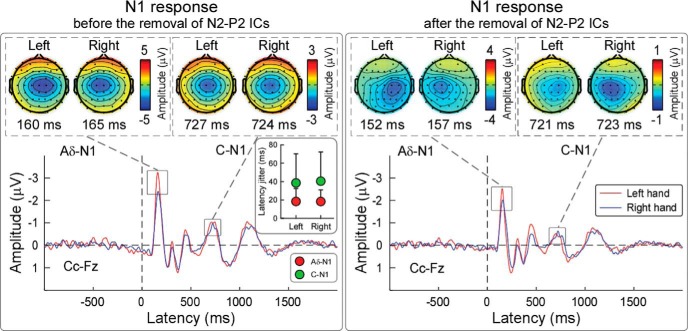
Detection of Aδ-N1 and C-N1 waves. Grand averages and scalp topographies of Aδ-N1 and C-N1 waves elicited by stimulating the hand dorsum on the left side (red waveforms) and the right side (blue waveforms). Left, Aδ-N1 and C-N1 waves are measured from the contralateral central electrode (Cc vs Fz), a montage that allows the isolation of Aδ-N1 and C-N1 peaks. The Aδ-N1 and C-N1 scalp topographies have a maximum contralateral to the stimulated side. Histograms in the inset show the absolute difference (mean ± SD) between single-subject and group-average latency of Aδ-N1 and C-N1 waves: the C-N1 wave has a larger jitter than the Aδ-N1 wave. Right, After the removal of the independent components capturing the activity of the partially overlapping N2–P2 vertex waves, the topographies of both the Aδ-N1 and C-N1 are more lateralized.

The left panel of Figure 4 shows the grand average LEP waveforms elicited by the stimulation of LH and RH at electrode Cc referenced to Fz. Using this Cc-Fz montage, both the Aδ-N1 and C-N1 waves could be clearly detected. Aδ-N1 and C-N1 scalp topographies showed a maximum over the central electrodes contralateral to the stimulated side. Across subjects, latencies and amplitudes of Aδ-N1 and C-N1 waves were as follows: (1) Aδ-N1: 167 ± 3 ms and −5.2 ± 0.3 μV (LH stimulation; mean ±SEM); 173 ± 2 ms and −4.3 ± 0.3 μV (RH stimulation); and (2) C-N1: 736 ± 6 ms and −3 ± 0.2 μV (LH stimulation); 737 ± 6 ms and −2.8 ± 0.2 μV (RH stimulation). The between-subject latency variability of the Aδ-N1 was 18 ± 2 ms (LH stimulation) and 19 ± 2 ms (RH stimulation). The between-subject latency variability of the C-N1 was 37 ± 4 ms (LH stimulation) and 40 ± 4 ms (RH stimulation). Two-way repeated-measures ANOVA conducted using the afferent pathway (two levels: Aδ and C) and the stimulated side (two levels: left and right) revealed a significant main effect of afferent pathway (*F*_(1,70)_ = 27.4, *p* < 0.001, η_p_^2^ = 0.28), indicating that the latency jitter was significantly larger for C-N1 than Aδ-N1 wave.

The right panel of Figure 4 shows the grand average LEP waveforms (Cc-Fz) after the removal of the N2-P2 complex using an additional ICA. The scalp topographies of both Aδ-N1 and C-N1 waves show that the negative maximum over the central electrodes contralateral to the stimulated side is more clearly isolated compared with the LEP waveforms before N2-P2 removal.

#### Relationship between C-N1 and other Aδ*-* and C-LEP waves

**Figure 5.**
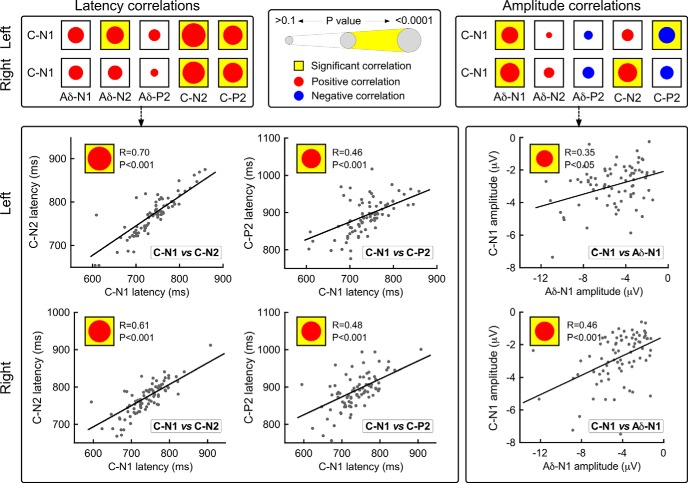
Correlations between C-N1 and other LEP waves. Top, All possible latency and amplitude correlations between the C-N1 and other LEP waves (i.e., Aδ-N1, Aδ-N2, Aδ-P2, C-N2, and C-P2). Red and blue dots represent positive and negative correlations and the dot size reflects the significance of the correlation. When a correlation was significant, the corresponding box was marked in yellow. Note that, for both LH and RH dorsum stimulations, C-N1 amplitudes were most strongly correlated with Aδ-N1 amplitudes. Bottom, Single-subject data for the significant correlations. Each dot represents a subject and black lines represent the best linear fit.

Results of correlation analysis between latency/amplitude of each C-LEP wave and latency/amplitude of the other Aδ-LEP and C-LEP waves are summarized in Figure 5 and Table 2. Some of these results are helpful in understanding the functional significance of C-LEPs in general and of the C-N1 wave in particular.

**Table 2.** Latency and amplitude correlations between LEP waves

		Latency correlations (*R* values)						Amplitude correlations (*R* values)					
		Aδ-N1	Aδ-N2	Aδ-P2	C-N1	C-N2	C-P2	Aδ-N1	Aδ-N2	Aδ-P2	C-N1	C-N2	C-P2
C-N1	Left	0.33	0.38*	0.18	—	0.70*	0.46*	0.35*	0.05	−0.10	—	0.18	−0.34*
	Right	0.26	0.26	0.08	—	0.61*	0.48*	0.46*	0.14	−0.17	—	0.44*	−0.22
C-N2	Left	0.20	0.15	0.11	0.70*	—	0.76*	0.13	0.56*	−0.43*	0.18	—	−0.49*
	Right	0.14	0.13	0.07	0.61*	—	0.76*	0.26	0.56*	−0.56*	0.44*	—	−0.69*
C-P2	Left	0.14	0.04	0.17	0.46*	0.76*	—	0.14	0.00	0.12	−0.34*	−0.49*	—
	Right	0.18	0.14	0.07	0.48*	0.76*	—	−0.12	−0.32*	0.42*	−0.22	−0.69*	—

Significant correlations for both LH and RH stimulations are shown in bold.

*p_fdr_ < 0.05.

First, the latencies of the different C-LEP waves (C-N1, C-N2, and C-P2) were strongly correlated among themselves, whereas they were not correlated with those of Aδ-LEP waves (Aδ-N1, Aδ-N2, and Aδ-P2) (Table 2). This finding confirms that Aδ-LEPs and C-LEPs are consequent to the arrival of somatosensory volleys relayed by different populations of myelinated and unmyelinated peripheral afferents.

Second, the amplitude of the C-N1 wave from both hands was significantly and positively correlated with that of the Aδ-N1 wave, whereas it was not consistently correlated with the amplitude of the other vertex waves: that is, C-N2, C-P2, Aδ-N2, and Aδ-P2 (*R* values and significance are reported in Table 2). In contrast, amplitude of the vertex C-N2 was significantly correlated with amplitudes of the vertex Aδ-N2, Aδ-P2, and C-P2, but not with the amplitude of either the Aδ-N1 or the C-N1 wave (*R* values and significance are reported in Table 2). This finding provides further evidence that the N1 waves elicited by both Aδ and C afferent volleys are generated by cortical structures different from those generating the vertex waves (Aδ-N2/P2 and C-N2/P2).

#### Dependence of human C-N1 amplitudes on stimulus energy and pain intensity

Amplitudes of the main Aδ-LEP and C-LEP waves for different stimulus energies (E1–E4) and hand stimulation sites (LH and RH), together with their statistical comparison, are summarized in Table 3. Two-way repeated-measures ANOVA with factors stimulus energy (four levels: E1–E4) and stimulated side (two levels: left and right) showed that Aδ-N1 amplitudes were significantly modulated by stimulus energy (*F*_(3,249)_ = 243.9, *p* < 0.001, η_p_^2^ = 0.86), stimulated side (*F*_(1,83)_ = 7.26, *p* = 0.009, η_p_^2^ = 0.08), and their interaction (*F*_(3,249)_ = 3.40, *p* = 0.02, η_p_^2^ = 0.06), and that amplitudes of the Aδ-N2, Aδ-P2, C-N1, C-N2, and C-P2 waves were significantly modulated only by stimulus energy.

**Table 3.** Effect of laser energy (E1–E4) and stimulation site (LH and RH) on LEP amplitudes

	LH stimulation (mean ± SEM)				RH stimulation (mean ± SEM)				Two-way ANOVA		
	E1	E2	E3	E4	E1	E2	E3	E4	Energy	Site	Interaction
Pain intensity (0–10)	3.4 ± 0.1	4.2 ± 0.1	5.7 ± 0.1	7.6 ± 0.1	3.3 ± 0.1	4.1 ± 0.1	5.5 ± 0.2	7.4 ± 0.2	*F* = 726.5***	*F* = 5.7*	*F* = 0.5
Aδ-N1	−0.3 ± 0.2	−0.9 ± 0.3	−5.3 ± 0.5	−11.2 ± 0.5	−0.1 ± 0.2	−1.2 ± 0.3	−4.0 ± 0.4	−9.5 ± 0.6	*F* = 243.9***	*F* = 7.3**	*F* = 3.4*
Aδ-N2	0.1 ± 0.3	−2.9 ± 0.6	−15.5 ± 1.3	−33.4 ± 1.4	−0.2 ± 0.4	−2.3 ± 0.5	−13.1 ± 1.1	−32.5 ± 1.4	*F* = 370.8***	*F* = 3.8	*F* = 1.6
Aδ-P2	0.8 ± 0.4	3.0 ± 0.7	13.3 ± 1.0	26.1 ± 1.2	−0.1 ± 0.5	2.8 ± 0.6	12.2 ± 1.0	25.9 ± 1.2	*F* = 300.7***	*F* = 1.9	*F* = 0.3
C-N1	−2.3 ± 0.3	−3.1 ± 0.2	−3.3 ± 0.2	−2.5 ± 0.2	−2.4 ± 0.3	−2.7 ± 0.3	−3.0 ± 0.3	−2.4 ± 0.2	*F* = 3.6*	*F* = 2.0	*F* = 0.4
C-N2	−6.4 ± 0.6	−9.3 ± 0.6	−8.5 ± 0.5	−6.2 ± 0.6	−6.4 ± 0.7	−9.3 ± 0.7	−8.5 ± 0.5	−7.7 ± 0.5	*F* = 9.6***	*F* = 1.1	*F* = 1.3
C-P2	6.1 ± 0.7	7.3 ± 0.6	4.0 ± 0.5	2.0 ± 0.8	6.2 ± 0.6	7.3 ± 0.6	5.3 ± 0.5	1.8 ± 0.5	*F* = 31.5***	*F* = 0.6	*F* = 1.0

Aδ-N2, Aδ-P2, C-N2, and C-P2 amplitudes (μV) were measured at Cz-average.

Aδ-N1 and C-N1 amplitudes (μV) were measured at Cc-Fz.

Significant effects are shown in bold.

*p < 0.05;

**p < 0.01;

***p < 0.001.

**Figure 6.**
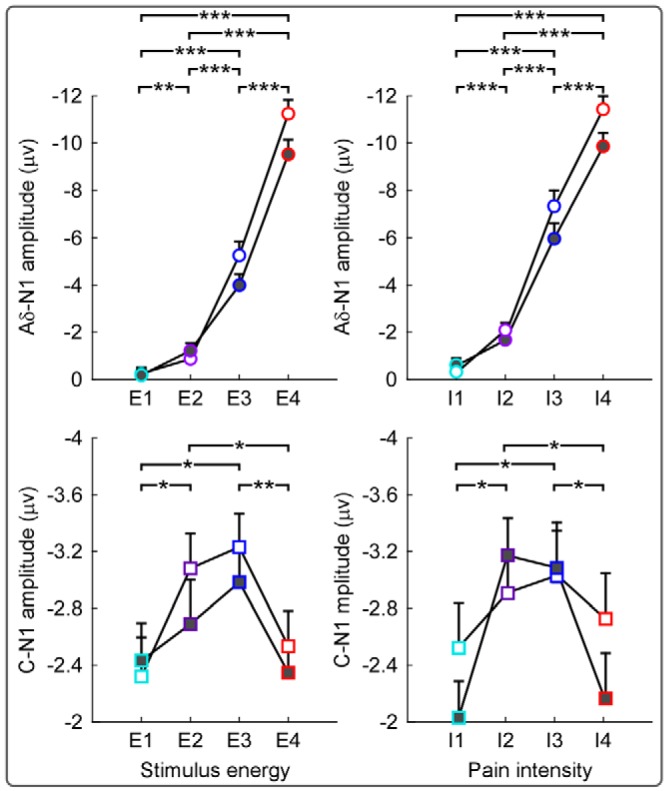
Dependence of human Aδ-N1 and C-N1 amplitudes on stimulus energy and perceived pain. Group-level amplitudes of Aδ-N1 (top, circles) and C-N1 (bottom, squares) waves elicited by stimulation of the left (open symbols) and right (solid symbols) hand dorsum at different levels of stimulus energy (left: E1–E4) and different categories of pain intensity (right: I1–I4). Error bars indicate SEM across subjects. Note that Aδ-N1 amplitudes increase monotonically with stimulus energy or pain intensity, whereas C-N1 amplitudes are maximal at moderate levels of stimulus energy (E2 and E3) or pain intensity (I2 and I3). *p < 0.05, **p < 0.01, ***p < 0.001.

*Post hoc* pairwise comparisons indicated that Aδ-N1 amplitudes at E3 and E4 were significantly larger than those at E1 and E2 (E1 < E3, E1 < E4, E2 < E3, E2 < E4, E3 < E4, *p* < 0.001 for all comparisons) for both stimulated sides, and Aδ-N1 amplitudes at E2 were significantly larger than those at E1 (E1 < E2, *p* < 0.01) for RH stimulation. In addition, pairwise comparisons showed that C-N1 amplitudes were significantly larger at E3 than at E1 and E4 (E3 > E1, *p* = 0.01; E3 > E4, *p* = 0.03), and at E2 than at E1 (E2 > E1, *p* = 0.03) for LH stimulation and larger at E3 than at E4 (*p* = 0.03) for RH stimulation. The results of these *post hoc* comparisons suggest that the higher the stimulus energy, the larger the Aδ-N1 amplitude (Fig. 6, top), whereas C-N1 amplitude is maximal at a moderate level of stimulus energy (i.e., E3; Fig. 6, bottom). Similarly, *post hoc* pairwise comparisons revealed that the higher the stimulus energy, the larger the amplitudes of Aδ-N2 and Aδ-P2 waves. In contrast, C-N2 and C-P2 amplitudes were maximal at a moderate level of stimulus energy (i.e., E2 or E3), as described previously (Hu et al., 2014b).

Two-way repeated-measures ANOVA with factors pain intensity (four levels: I1–I4) and stimulated side (two levels: left and right) showed that Aδ-N1 amplitudes were significantly modulated by pain intensity (*F*_(3,240)_ = 240.8, *p* < 0.001, η_p_^2^ = 0.72) and stimulated side (*F*_(1,80)_ = 5.30, *p* = 0.024, η_p_^2^ = 0.06), while C-N1 amplitudes waves were significantly modulated only by pain intensity (*F*_(3,213)_ = 3.59, *p* = 0.015, η_p_^2^ = 0.05).

*Post hoc* pairwise comparisons indicated that Aδ-N1 amplitudes monotonically increased with perceived pain at both stimulation sites (I1 < I2 < I3 < I4, *p* < 0.001 for all comparisons) and that C-N1 amplitudes were significantly larger at I2 and I3 than at I1 and I4 (I1 < I2, I4 < I2, I1 < I3, I4 < I3, *p* < 0.05 for all comparisons) for RH stimulation.

### Experiment 3: Rat ECoG

#### Waveforms and topographies of the early part of rat C-LEPs

**Figure 7.**
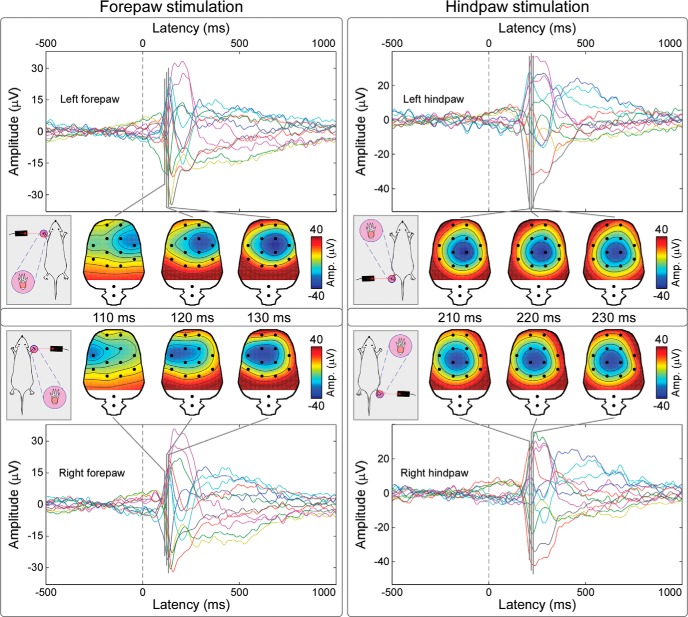
S1 contribution to the earliest part of rat C-LEPs elicited by stimulation of the left and right (top and bottom) forepaw and hindpaw (left and right). Grand average waveforms from all electrodes are plotted in different colors and superimposed. Series of scalp topographies of the earliest part of C-LEPs (110–130 ms, forepaw; 210–230 ms, hindpaw) are displayed at 10-ms intervals. Note that the scalp topographies of the earliest part of the C-LEPs elicited by forepaw stimulation display a negativity strongly contralateral to the stimulated side, whereas the scalp topographies of the earliest part of C-LEPs elicited by hindpaw stimulation, albeit contralateral, are more centrally distributed. These topographies are compatible with the somatotopical organization of the S1, and indicate that S1 contributes to the early part of the brain response evoked by C-fiber stimulation.

Figure 7 shows the grand average rat C-LEPs after stimulation of the forepaw and hindpaw on the right and left sides. Scalp topographies of the earliest activity in the response evoked by right and left forepaw stimulation (110–130 ms; Fig. 7, left) displayed a clear negative maximum on the hemisphere contralateral to the stimulated side. Scalp topographies of the earliest part of the response elicited by right and left hindpaw stimulation (210–230 ms; Fig. 7, right) were more centrally distributed.

#### Isolation of the C-N1 wave

**Figure 8.**
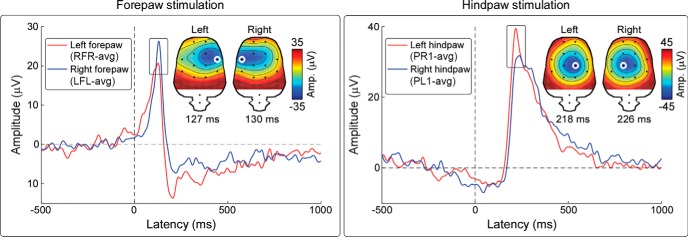
Isolation of C-N1 waves in rats. Grand-average C-N1 waves elicited by the stimulation of the left (red waveforms) and right (blue waveforms) forepaw (left panel) and hindpaw (right panel). The C-N1 was optimally detected from the electrodes highlighted in white: RFR after left forepaw stimulation, LFL after right forepaw stimulation, PR1 after left hindpaw stimulation, and PL1 after right hindpaw stimulation. Scalp topographies at peak latency show a maximum contralateral to the stimulated side, clearly lateralized in the response elicited by forepaw stimulation and less lateralized in the response elicited by hindpaw stimulation.

Similar to human Aδ- and C-LEP data, the widespread scalp distribution of the main vertex C-LEP components makes the detection of the early lateralized neural activities as a separate peak difficult (Xia et al., 2016). To reduce the influence of widespread activities and thus enhance spatially discrete activities, we average referenced the data (Bertrand et al., 1985). This allows to visualize the first two negative waves (N1, N2) and the third positive wave (P2) as separate deflections (Xia et al., 2016). The N1 wave was optimally detected from electrode RFR (after left forepaw stimulation), LFL (after right forepaw stimulation), PR1 (after left hindpaw stimulation), and PL1 (after right hindpaw stimulation) (Fig. 8). Peak latency and amplitude of the N1 wave were as follows: 125 ± 11 ms, −35.7 ± 6.2 μV (left forepaw); 125 ± 3 ms, −29.3 ± 6.0 μV (right forepaw); 230 ± 7 ms, −42.0 ± 6.5 μV (left hindpaw); and 230 ± 6 ms, −34.3 ± 5.4 μV (right hindpaw).

#### Dependence of rat C-N1 amplitudes on stimulus energy

**Figure 9.**
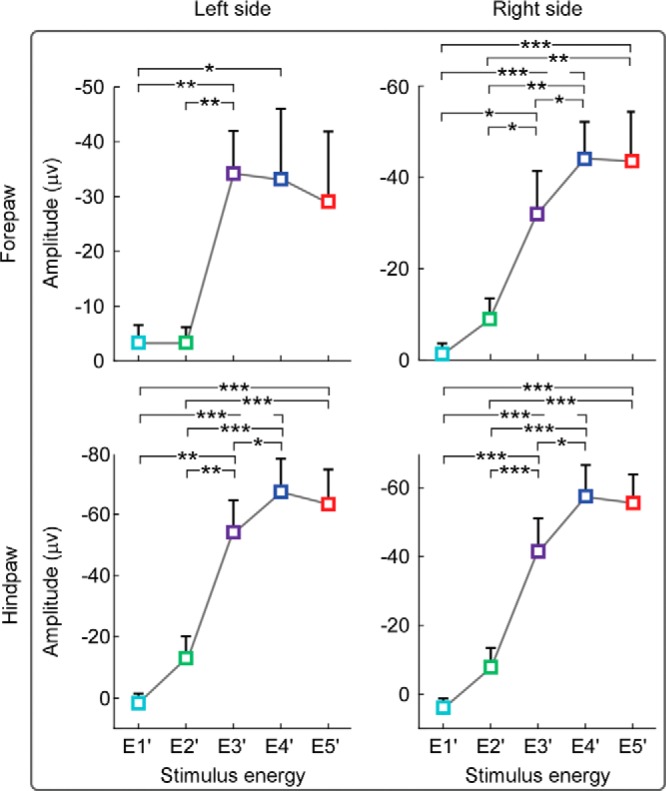
Dependence of rat C-N1 amplitudes on stimulus energy. Group-level amplitudes of C-N1 waves elicited by stimulation of the left and right forepaw (top) and hindpaw (bottom) at different levels of stimulus energy (E1′–E5′). Error bars indicate SEM across subjects. Note that, whereas human C-N1 amplitude is maximal at moderate level of stimulus energy (Fig. 6), rat C-N1 amplitude increases quasimonotonically with stimulus energy and only plateaus at E4′–E5′. *p < 0.05, **p < 0.01, ***p < 0.001.

In striking contrast to the human data, the amplitude of the rat C-N1 displayed a monotonic relationship with stimulus energy (Fig. 9). Two-way repeated-measures ANOVA with factors stimulus energy (five levels: E1'-E5') and stimulated side (four levels: left and right forepaw and hindpaw) showed that C-N1 amplitudes were significantly modulated by stimulus energy (*F*_(4,44)_ = 41.41, *p* < 0.001, η_p_^2^ = 0.79), but not by stimulated side (*F*_(3,33)_ = 1.93, *p* = 0.14, η_p_^2^ = 0.15) or their interaction (*F*_(12,132)_ = 1.64, *p* = 0.09, η_p_^2^ = 0.11). *Post hoc* pairwise comparisons revealed that C-N1 amplitudes at E3′, E4′, and E5′ were significantly larger than those at E1′ and E2′ (*p* < 0.05; Fig. 9), indicating a strong positive relationship between stimulus energy and C-N1 amplitude.

## Discussion

### Main findings

We describe for the first time the somatotopical organization and the stimulus–response functions of the electrocortical responses elicited in the S1 by selective stimulation of small-myelinated Aδ and unmyelinated C afferents in humans and rats. We obtained five main results.

First, both in human and rat recordings, the topographical distribution of the earliest part of the cortical response elicited by C-fiber input (C-LEPs) was clearly dependent on the stimulated limb (Figs. 2, 7). When the stimulus was delivered to the hand or the forepaw, the response displayed a negative maximum in the hemisphere contralateral to the stimulation side. In contrast, when the stimulus was delivered to the foot or the hindpaw, the response was more centrally distributed (Figs. 2, 7).

Second, source analysis of human EEG confirmed that the cortical sources of the earliest part of C-LEPs were located in the postcentral gyrus, in positions compatible with the representations of the stimulated body district within S1 (Fig. 3).

Third, in human EEG, a bipolar Cc-Fz montage allowed detecting this S1 activity as a distinct early deflection (C-N1 wave), peaking at 710–730 ms after hand stimulation (Fig. 4). In contrast, a C-N1 could not be clearly isolated after foot stimulation because of the more medial foot representation within S1 results in a strong spatial and temporal overlap with the large amplitude C-N2 (Fig. 1). Different from human recordings, in rat ECoG a clear C-N1 could be isolated after both forepaw and hindpaw stimulation (Fig. 8), because of the different representation of the body surface within S1.

Fourth, stimulus–response functions were different in humans and rats. In humans, C-N1 had maximal amplitude at moderate stimulus energy and pain intensity (Fig. 6), whereas in rats C-N1 amplitude increased quasimonotonically with stimulus energy (Fig. 9). The difference in stimulus–response functions was caused by the combination of the across-species difference in thermal sensitivity of Aδ nociceptors (Hu et al., 2015) with the dependence of these electrocortical responses in the time domain on stimulus relevance (Iannetti et al., 2008; Mouraux and Iannetti, 2009).

Finally, there was a decoupling between the activity of the S1 (indexed by C-N1 in human recordings) and the activity of the supramodal generators of N2-P2 vertex waves (Mouraux and Iannetti, 2009): the C-N1 amplitude was positively correlated with the amplitude of the Aδ-N1 wave, but not correlated with the amplitude of the other vertex waves: C-N2, C-P2, Aδ-N2, and Aδ-P2 (Fig. 5). This finding highlights the functional dissociation between the generators of somatosensory-specific (C-N1 and Aδ-N1) and supramodal waves (Aδ-N2/P2 and C-N2/P2).

### Detecting C-fiber responses in the S1 of humans and rodents

Stimulus–response functions of C-fiber responses in the S1 of rats and humans were clearly different. Whereas in humans the C-N1 wave was maximal at moderate levels of stimulus energy and pain intensity (Fig. 6), in rats it increased monotonically with stimulus energy and only plateaued at the highest stimulus energy (Fig. 9). To understand this difference, it is important to consider two physiological aspects: (1) the functional significance of the electrocortical responses elicited by sudden and fast-rising sensory stimuli and (2) the different heat sensitivity of Aδ skin nociceptors in rodents and humans.

The large brain potentials detected in EEG and ECoG recordings in response to sudden sensory stimuli do not always faithfully reflect the intensity of the afferent peripheral input; instead, they are strongly determined by contextual factors such as stimulus saliency (Iannetti et al., 2008; Mouraux and Iannetti, 2009; Ronga et al., 2013). This simple concept explains why, in humans, the C-fiber response (e.g., the C-N1; Figs. 1, 2, 3, 4) is larger when preceded by an Aδ-fiber response elicited by stimuli of moderate energy and pain intensity (E2–E3, I2–I3; Fig. 6), whereas it becomes smaller when preceded by a large Aδ-fiber response elicited by stimuli of high energy (E4) (Hu et al., 2014b). For this reason, the C-fiber response has for a long time been considered to be detectable only when the concomitant activation of Aδ-fibers is avoided or reduced (Bromm and Treede, 1983; Bragard et al., 1996; Mouraux et al., 2003).

This reasoning does not apply to the C-fiber ECoG responses measured in rats because Aδ-nociceptors have different heat sensitivity in rodents and humans. In rats, as demonstrated by recordings from peripheral axons and spinal cord neurons (Devor et al., 1982; Lynn and Shakhanbeh, 1988; Sikandar et al., 2013), estimates of conduction velocity of peripheral afferents (Jiang et al., 2007), and nocifensive behaviors (Fan et al., 2009), Aδ units are virtually insensitive to heat. For this reason, in rats, there is no Aδ-fiber response to heat, and the ECoG response elicited by the C-fiber afferent volley is monotonically graded with the energy of the applied stimulus (Fig. 9).

### Unmyelinated nociceptive input elicits somatotopically organized responses in S1

Although strongly debated only a few years ago (Bushnell et al., 1999; Ploner et al., 2002), there is now compelling evidence that nociceptive stimulation elicits robust responses in the S1. To settle the issue of whether S1 responds to nociceptive input, the use of somatosensory stimuli that activate Aδ- and C-nociceptors selectively, that is, without the coactivation of non-nociceptive Aβ-fibers (Baumgärtner et al., 2005), has been decisive. Psychophysically, selective stimulation of Aδ-afferents has allowed revealing that first pain and touch have remarkably similar spatial acuity across the body (Mancini et al., 2013). The same Aδ-selective laser stimulation has allowed the identification of somatosensory-specific deflections in the human EEG, from the seminal evidence that S1 contributes to the N1 wave (Tarkka and Treede, 1993) to the comprehensive characterization of N1 topography and underling sources (Valentini et al., 2012; Hu et al., 2014b). Finally, phase-encoded fMRI combined with the same laser stimuli has allowed demonstrating the existence of fine-grained maps of individual digits in the human S1 (Mancini et al., 2012).

Whether and how S1 also responds to unmyelinated C-fiber input remains an open issue. Traditionally, second pain consequent to the arrival of the afferent volley transmitted by slow-conducting C-units is considered to be less well localized than the Aδ-mediated first pain, although rigorous studies of spatial acuity for second pain are lacking. Some investigators have suggested that C input elicits responses in the area 3a of S1 (Vierck et al., 2013), in contrast to the responses to Aδ input, which are localized in areas 3b/1 (Whitsel et al., 2009; Vierck et al., 2013). However, these studies used long thermal stimuli delivered through mechanical probes that unavoidably coactivate touch-related Aβ-afferents. It is therefore difficult to rule out that these S1 responses are contributed by the concomitant tactile input: indeed, as detailed previously (Nahra and Plaghki, 2005), even if the applied tactile input is constant, the heating of the thermode can cause a transient response still mediated by the tactile input.

Here, we used topographical analysis and source modeling of human EEG and rat ECoG data to provide converging and across-species evidence that the earliest part of the cortical response elicited by C-fiber stimulation is generated in the S1 contralateral to the stimulated side, and that this response follows the representation of the body surface within S1 (Penfield and Boldrey, 1937; Kandel et al., 2013). The observation that C-N1 amplitude is maximally correlated with the amplitude of the Aδ-N1 wave, but not with the amplitude of the other vertex waves (Aδ-N2/P2 and C-N2/P2; Fig. 5), provides additional evidence that the cortical generator of C-N1 is similar, if not identical, to that of Aδ-N1, which has been repeatedly shown to be largely contributed by the contralateral S1 (Treede et al., 1988; Valentini et al., 2012; Hu et al., 2014a). However, the limited spatial resolution of both ECoG and scalp EEG (Buzsáki et al., 2012; Gratiy et al., 2017) does not allow excluding the possibility that the C-N1 response (as well as the Aδ-N1 response; Treede et al., 1988; Valentini et al., 2012) is contributed by sources in the primary motor cortex, whose topographical arrangement follows closely that of S1 (Penfield and Boldrey, 1937). Direct recording of local field potentials is a more adequate approach to address this issue.

C-fiber afferents are the phylogenetically oldest of the somatosensory system (Franzén et al., 1996; van Bijsterveld et al., 2003). Their main function is to inform the CNS of potentially noxious environmental stimuli (Julius and Basbaum, 2001). It is therefore unsurprising that the C-fiber conveys at least some information about the spatial location of noxious stimulation across the body surface also considering that even the lateral periaqueductal gray receives somatotopically organized nociceptive projections (Holstege et al., 1996; Benarroch, 2012). The somatotopically organized projections to the S1 are likely to be a prerequisite for deploying spatially-coherent defensive moments. Although the present results provide compelling evidence for a gross somatotopy within S1, the resolution of the spatial acuity of the C-fiber system remains to be fully characterized.
